# Long‐term impact of type 2 diabetes onset on dementia incidence rate among New Zealanders with impaired glucose tolerance: A tapered‐matched landmark analysis over 25 years

**DOI:** 10.1002/alz.13855

**Published:** 2024-06-14

**Authors:** Dahai Yu, Zheng Wang, Uchechukwu Levi Osuagwu, Karen Pickering, John Baker, Richard Cutfield, Yamei Cai, Brandon J. Orr‐Walker, Gerhard Sundborn, Bingjie Qu, Zhanzheng Zhao, David Simmons

**Affiliations:** ^1^ Department of Nephrology the First Affiliated Hospital, Zhengzhou University Zhengzhou China; ^2^ Primary Care Centre Versus Arthritis School of Medicine Keele University Keele UK; ^3^ Translational Health Research Institute (THRI) Western Sydney University Sydney New South Wales Australia; ^4^ School of Medicine Western Sydney University Sydney New South Wales Australia; ^5^ Diabetes Foundation Aotearoa Otara Auckland New Zealand; ^6^ Department of Diabetes and Endocrinology Counties Manukau Health Otahuhu Auckland New Zealand; ^7^ Department of Diabetes and Endocrinology Waitemata District Health Board Takapuna Auckland New Zealand; ^8^ Section of Pacific Health the University of Auckland Auckland New Zealand

**Keywords:** dementia, impaired glucose tolerance, landmark analysis, tapered matching, type 2 diabetes

## Abstract

**INTRODUCTION:**

We aimed to investigate the association between the onset of type 2 diabetes (T2D) and dementia incidence rates (IR) in the population with impaired glucose tolerance (IGT) identified in primary care in New Zealand (NZ) over 25 years.

**METHODS:**

Tapered matching and landmark analysis (accounting for immortal bias) were used to control for potential effects of known confounders. The association between T2D onset and 5‐ and 10‐year IR of dementia was estimated by weighted Cox models.

**RESULTS:**

The onset of T2D was significantly associated with the 10‐year IR of dementia, especially in the socioeconomically deprived, those of non‐NZ European ethnicity, those currently smoking, and patients with higher metabolic measures.

**DISCUSSION:**

Our findings suggest that the onset of T2D is a significant risk factor for dementia in individuals with IGT. Dementia screening and structured diabetes prevention are vital in the population with IGT, particularly those from deprived or ethnic minority backgrounds.

**Highlights:**

- Increased dementia incidence rate links with T2D onset in people with IGT.
- Significant incidence varied by ethnicity, socioeconomic status, and health factors.
- Results emphasize the diabetes manage and socioeconomic factors on dementia risk.
- Secondary analysis highlights the key role of vascular health in dementia prevention.

## 1 BACKGROUND

Dementia poses a significant burden on global public health, and strong evidence links diabetes to an increased risk of developing dementia.^1^, ^2^, ^3^ In New Zealand (NZ), the prevalence of dementia reached 4% in the general population aged 60 years, with significantly higher rates in non‐NZ European ethnicities (5.4%–6.3% for minority ethnic groups) compared to 3.7% for those of NZ European descent.^4^ This increased risk is associated with a significantly higher health economic burden and loss of healthy life expectancy.^5^

Epidemiological studies suggest that diabetes, particularly type 2 diabetes (T2D), may increase the risk of dementia in the general population,^6^, ^7^, ^8^ indicating that chronic hyperglycemia could increase vulnerability to dementia. Observational studies also suggest an association between prediabetes, an intermediate mild degree of hyperglycemia, and increased dementia risk.^9^, ^10^ However, a recent systematic review revealed conflicting results, suggesting that prediabetes may not independently elevate dementia risk compared to normoglycemia, with potential influences from different prediabetes definitions.^11^

A retrospective cohort study in the United States, incorporating 2330 individuals with prediabetes defined by hemoglobin A1C (HbA1c), of whom 1040 developed T2D during follow‐up, found an association between T2D onset and increased dementia risk.^12^ Prediabetes, a condition indicating a high risk of developing diabetes, includes subtypes such as impaired glucose tolerance (IGT)—a state where blood glucose levels are higher than normal but not high enough for a diabetes diagnosis, typically identified through an oral glucose tolerance test (OGTT). However, it remains unclear if the mild hyperglycemia of IGT is associated with an increased dementia risk upon T2D onset, given the discrepancies between OGTT and HbA1c and the low sensitivity of HbA1c in identifying patients with IGT.^13^ Additionally, modifiable risk factors such as smoking, socioeconomic deprivation, hypertension, and obesity have been identified as contributors to dementia risk.^14^, ^15^ It is not yet clear whether the association between T2D onset and dementia incidence rate differs in the IGT population with or without these modifiable risk factors, which could inform targeted screening efforts. Furthermore, it would be valuable to understand if the association between T2D onset and dementia incidence rate varies across different ethnicity backgrounds, considering the higher prevalence of dementia among individuals from minority ethnic groups in NZ.

To investigate these questions, we conducted a prospective cohort study based on landmark analysis (controlling for immortal bias) comparing the 5‐ and 10‐year rate of incident dementia between individuals with and without T2D onset in the IGT population. We matched conventional risk factors using a cohort derived from longitudinal prediabetes audit data linked with national registration databases in NZ.

## 2 METHODS

### 2.1 Data setting

The Diabetes Care Support Service (DCSS) was established in 1991 to enhance diabetes care in West, East, and South Auckland, NZ, through general practice audits.^16^ As part of its operations, the DCSS collected data on individuals with IGT. To conduct this study, we assembled a cohort of patients aged 18 years and above with IGT by linking the de‐identified DCSS database with an extensive range of nationally accessible databases, including not only national death registration, and hospitalization but also primary care records, outpatient records, cancer registry, pharmaceutical claim, and socioeconomic status data. This comprehensive approach to data linkage significantly broadens the scope of our study, enhancing the robustness of our findings by ensuring a broader capture of patient data across various settings.

IGT was diagnosed based on a 2‐h glucose level of 7.8–11.0 mmol/L on an OGTT.^17^ The final dataset comprised demographics, clinical measurements (smoking, blood pressure [BP], body mass index [BMI], HbA1c, and lipids), and treatment details (e.g., antihypertensive, statin, antiplatelet, and/or anticoagulant treatment). We ensured data quality through internal quality control policies and audits.^18^, ^19^ To cross‐validate the prescription data in DCSS, we utilized pharmaceutical claims data from 2006 onwards, as National Health Index numbers were not universally implemented before that time. We included data for all patients from their first DCSS enrollment date until their last enrollment on July 31, 2018.

The North Health Ethics Committee approved the use of DCSS data for research purposes in 1992 and as an ongoing audit in 1996 (92/006). On March 25, 2019, the need for ethics review was waived. Anonymized data were used for this analysis, and signed consent was obtained from an authorized signatory for each general practice. This manuscript complies with the Strengthening the Reporting of Observational Studies in Epidemiology (STROBE) reporting guideline.

### 2.2 Exposure

In this study, we identified patients with IGT and classified them based on their exposure to T2D. Exposure was defined as newly diagnosed T2D recorded in any of the comprehensively linked datasets, including but not limited to hospitalization records, primary care records, outpatient records, and pharmaceutical claims. To investigate the impact of T2D onset on dementia incidence rate, we performed a landmark analysis, selecting a fixed time after cohort entry for conducting survival analysis. Only patients with IGT who were alive at the landmark date were included, and T2D onset was determined by exposure before the landmark date. The exposure window, ranging from the index date to the landmark time point, was used to evaluate the exposure. Subsequently, the outcome was assessed from the landmark time point. We predetermined five landmark time points, specifically at 1, 2, 3, 4, and 5 years after the cohort enrollment date. Exposure status was assigned to patients with IGT who remained alive at the landmark dates. The methodology for the landmark analysis is depicted in Figure S1.

### 2.3 Outcome

In this study, the primary outcome of interest was incident dementia. Incident dementia was defined as the first documented case of dementia recorded in the linked datasets during the follow‐up period after the landmark time point, utilizing a broad spectrum of data sources to ensure comprehensive capture. This approach aimed to mitigate potential information bias. Participants with IGT were followed up until an outcome of interest occurred or until December 31, 2019, for those who did not experience any outcome of interest. The identification of outcomes relied on primary International Classification of Diseases, Ninth Revision (ICD‐9) and ICD‐10 codes (Table S1).

**RESEARCH IN CONTEXT**

1. **Systematic review**: We conducted a review of relevant literature using PubMed. The association between the risk of dementia in individuals with prediabetes showed heterogeneity, possibly influenced by the onset of type 2 diabetes (T2D). However, the specific investigation of the link between T2D development and dementia incidence in individuals with impaired glucose tolerance (IGT) is lacking.
2. **Interpretation**: Our study employed innovative landmark analysis and tapered matching strategies to establish large comparative cohorts of individuals with IGT (*N* = 26,794) in New Zealand. The results indicated that individuals with IGT who later developed T2D faced a significantly higher long‐term incidence of dementia. Subgroup analysis underscored the importance of targeted preventive measures, such as lifestyle modification, smoking cessation, and screening strategies, tailored to specific high‐risk subgroups within the IGT population. Secondary analysis on vascular dementia emphasizes the importance of vascular health in dementia prevention.
3. **Future directions**: Our findings suggest that the association between IGT and long‐term dementia incidence is influenced by the onset of T2D. Targeted preventive interventions are urgently needed, especially for high‐risk subgroups within the population with IGT. Implementing diabetes screening programs and promoting lifestyle changes have the potential to reduce the burden of dementia among individuals with IGT.

### 2.4 Covariates

In this analysis, we considered several potential confounding factors as covariates. These included patient demographic characteristics (age, sex, ethnicity), lifestyle factors (smoking status), and clinical measurements such as BMI, BP, HbA1c, lipids, and estimated glomerular filtration rate (eGFR). Additionally, we accounted for the presence of treatments such as antihypertensive, anticoagulant, and lipid‐lowering drugs by index date. To assess the socioeconomic status of participants, we utilized the NZDep2013 Index of Deprivation, which assigns an Index of Multiple Deprivation (IMD) score to each meshblock in New Zealand based on the distribution of the first principal component scores.^20^ The IMD score ranges from 1 to 10, with lower scores indicating lower levels of deprivation. To ensure sufficient statistical power, we categorized the IMD into five groups: IMD‐1 (least deprived: NZDep2013 scores of 1–2), IMD‐2, IMD‐3, IMD‐4 (scores of 3–4, 5–6, and 7–8, respectively), and IMD‐5 (most deprived: scores of 9–10). These categorizations were consistent with previous deprivation measures.

The DCSS was a key component of our study, operational from 1994 to July 2018. It targeted patients with IGT who were also registered with primary care settings in NZ. Throughout the study period, all participants received continuous services from the DCSS through their primary care provider. The program's cessation in July 2018 did not impact our study's data collection or the outcomes analysis. The data for outcomes were independently extracted from national registration databases, ensuring their validity. Censoring in our study was conducted only in cases of death or when the outcome of interest occurred, not based on the discontinuation of services from the DCSS.

### 2.5 Statistical analysis

To address potential confounding factors, we employed tapered matching techniques in our analysis.^21^ This method compared the incidence rates of dementia between two groups: the focal group (IGT with T2D onset during the exposure time window) and the control group (IGT without T2D onset during the exposure time window). Entropy balancing was used to gradually match the control cohort with the focal cohorts using additional covariates, observing how the matched cohort changed in terms of incidence rate ratio (IRR) and unmatched covariates.

To minimize model dependence and imbalances between comparative groups, we used coarsened exact matching (CEM) before tapered matching and balancing.^22^, ^23^ Ten matching steps were performed for each of the 5 years of the landmark analysis, and patients with IGT in the comparative groups were retained if matched in the 10th step (Figure S2–S6).

Our analysis focused on participants with areas of common support and used entropy balancing to minimize differences in matching variable distribution between comparison groups. This involved reweighting the unexposed group by directly incorporating covariate balance into the weight function at each matching step, aiming to achieve key target moments like mean, variance, and skewness. All pre‐processing was conducted without reference to outcomes. The final cohort refers to the cohort established after the CEM process, at which point the size of the cohort and the outcome counts remain unchanged. The final tapered‐matched model, on the other hand, denotes the weighted model that applies weights derived from entropy matching within the final cohort.

In order to provide a comprehensive view of the age distribution within our cohort at the outset and throughout the follow‐up period, the actual age by temporal median and interquartile range (IQR) at index date, landmark timepoints, and subsequent follow‐up time points across different landmark analyses are presented in Figure S7.

Weighted Cox proportional hazards regression, incorporating matching weights estimated from entropy balancing in each step, was used to account for the competing risk of all‐cause death (except deaths due to incident dementia). The analysis estimated the IRR of dementia between the comparison groups. Missing data were minimal: for each of covariates including BMI, BP, HbA1c, lipids, and eGFR, less than 1% of the records were missing among eligible participants. In instances where 5.3% of participants had one or more missing covariates, we addressed this by creating six imputed datasets. These datasets were derived using multiple imputations with chained equations, adhering to Rubin's rules for combining multiple imputations.

The crude incidence rates were calculated using a Poisson regression model within the final cohort, following the CEM process, without the application of weights (Table S2). The weighted incidence rates were derived from weighted Cox regression models, incorporating weights to balance covariates, within the final cohort (Table S2).

Subgroup analysis was conducted to investigate differential effect of T2D onset and the incidence rate of dementia across various predefined subgroups (sex, age‐group, NZE, deprivation status, smoking status, obesity, and levels of clinical measurements [systolic blood pressure {SBP}, total cholesterol {TC}, low density lipoprotein {LDL}, and eGFR]). These analyses utilized 5‐year landmark models to include a test of interaction to determine whether there were significant differences in the effect of T2D onset on dementia incidence rate. Significant *P*‐values for interaction in these tests would indicate heterogeneity in the effect across subgroups.

A secondary analysis was performed to estimate both the crude and weighted 5‐year and 10‐year incidence rates of vascular dementia in patients with and without T2D onset, across 1‐ to 5‐year landmarks. Additionally, we evaluated the adjusted IRR for the association between T2D onset and the incidence of vascular dementia, both overall and within specific subgroups, using the final model based on the 5‐year landmark. All analyses were performed using Stata/MP version 18.0 (StataCorp LLC), and statistical significance was set at *P* < 0.05 (two‐tailed).

## 3 RESULTS

A total of 26,794 patients with IGT were initially included in the DCSS from 1994 to 2018. Participants with a history of outcomes, death, or loss of follow‐up between the enrollment date and the landmark time point were excluded. Through 10 matching steps, we created matched cohorts of patients with and without the onset of T2D for 1‐, 2‐, 3‐, 4‐, and 5‐year landmark analysis (Figure S2–S6). The number of participants in the exposed group was 157, and in the unexposed group, there were 2000 participants for the 1‐year analysis. For the 2‐year analysis, the numbers were 329 versus 3925, for the 3‐year analysis, 505 versus 5156, for the 4‐year analysis, 597 versus 5051, and for the 5‐year analysis, 650 versus 4352 controls, respectively.

Table S3 and Table 1 present the characteristics of individuals with IGT, both with and without the onset of T2D, before and after matching. After the tapered matching, particularly using entropy matching, there were no significant differences observed in the variables included in the matching process between patients with IGT with and without the onset of T2D for all landmark analyses (Table 1). This successful matching suggests that the matching technique effectively balanced the covariates, ensuring comparability between the exposed and unexposed groups.

**TABLE 1:** Comparison of patients with and without onset of type 2 diabetes in patients with impaired glucose tolerance in the final matched cohorts.

Parameter	1‐Year landmark			2‐Year landmark			3‐Year landmark			4‐Year landmark			5‐Year landmark		
	Without T2D onset	With T2D onset	*P*‐Value	Without T2D onset	With T2D onset	*P*‐Value	Without T2D onset	With T2D onset	*P*‐Value	Without T2D onset	With T2D onset	*P*‐value	Without T2D onset	With T2D onset	*P*‐Value
	2000	157		3925	329		5156	505		*N* = 5051	*N* = 597		*N* = 4352	*N* = 650	
Age, years	57.2 (13.3)	57.2 (12.6)	0.607	57.1 (12.5)	58.6 (11.5)	0.111	57.7 (12.4)	58.1 (12.2)	0.085	56.6 (12.4)	57.5 (12.1)	0.076	56.5 (12.0)	57.2 (12.3)	0.342
Female gender, % (SE)	62.5 (0.03)	62.8 (0.05)	0.965	54.5 (0.02)	54.6 (0.04)	0.975	51.6 (0.01)	51.6 (0.03)	0.979	53.1 (0.01)	53.1 (0.03)	0.981	52.7 (0.01)	52.7 (0.02)	0.983
New Zealand European, % (SE)	47.8 (0.03)	47.8 (0.05)	0.990	45.9 (0.02)	45.9 (0.04)	0.993	44.2 (0.01)	44.2 (0.03)	0.994	44.0 (0.01)	44.0 (0.03)	0.995	44.0 (0.01)	44.0 (0.02)	0.995
Enrolled cohort, % (SE)															
1994–1998	1.2 (0.01)	2.1 (0.01)	0.578	0.6 (0.004)	1.0 (0.01)	0.814	0.6 (0.003)	0.7 (0.005)	0.999	0.8 (0.01)	0.7 (0.01)	0.874	0.8 (0.01)	0.5 (0.03)	0.676
1999–2003	8.2 (0.02)	4.3 (0.02)		7.9 (0.02)	6.1 (0.02)		7.0 (0.01)	6.8 (0.01)		6.2 (0.01)	7.0 (0.01)		6.5 (0.01)	8.1 (0.01)	
2004–2008	14.9 (0.02)	20.2 (0.04)		16.0 (0.02)	18.4 (0.03)		15.5 (0.01)	15.5 (0.02)		15.8 (0.01)	14.1 (0.02)		18.5 (0.01)	16.1 (0.02)	
2009–2013	46.9 (0.03)	43.6 (0.05)		45.4 (0.02)	43.9 (0.04)		46.6 (0.01)	46.6 (0.03)		52.1 (0.01)	53.4 (0.03)		60.8 (0.02)	62.4 (0.02)	
2014–2018	28.8 (0.02)	29.8 (0.05)		30.2 (0.01)	30.6 (0.03)		30.3 (0.01)	30.4 (0.03)		25.0 (0.01)	24.7 (0.02)		13.3 (0.01)	12.9 (0.02)	
IMD group (NZDep13 scale), % (SE)															
Least deprivation: IMD‐1 (1 or 2)	7.1 (0.01)	7.4 (0.03)	0.983	7.4 (0.01)	7.1 (0.02)	0.978	8.7 (0.01)	8.8 (0.02)	0.998	9.7 (0.01)	9.9 (0.02)	0.920	9.0 (0.01)	9.0 (0.01)	0.999
IMD‐2 (3 or 4)	13.4 (0.02)	11.7 (0.03)		16.0 (0.01)	16.8 (0.03)		16.4 (0.01)	15.9 (0.02)		15.6 (0.01)	14.6 (0.02)		15.8 (0.01)	15.7 (0.02)	
IMD‐3 (5 or 6)	12.9 (0.03)	14.9 (0.04)		14.7 (0.02)	13.3 (0.02)		11.2 (0.01)	11.8 (0.02)		11.0 (0.01)	12.5 (0.02)		11.5 (0.01)	11.5 (0.02)	
IMD‐4 (7 or 8)	20.0 (0.02)	19.1 (0.04)		15.1 (0.01)	16.3 (0.03)		15.4 (0.01)	15.2 (0.02)		15.4 (0.01)	14.6 (0.02)		15.8 (0.01)	15.9 (0.02)	
Most deprivation: IMD‐5 (9 or 10)	46.6 (0.03)	46.8 (0.05)		46.7 (0.02)	46.4 (0.04)		48.3 (0.01)	48.3 (0.03)		48.2 (0.01)	48.4 (0.03)		47.9 (0.01)	47.9 (0.02)	
Smoking status, % (SE)															
Never smoking	55.6 (0.03)	55.3 (0.05)	0.994	51.2 (0.02)	51.0 (0.04)	0.998	53.5 (0.02)	53.4 (0.03)	0.999	53.2 (0.01)	53.1 (0.03)	0.999	53.1 (0.01)	53.0 (0.02)	0.999
Ex‐smoker	27.1 (0.03)	27.7 (0.05)		33.4 (0.02)	33.7 (0.03)		30.9 (0.01)	31.1 (0.03)		30.0 (0.01)	30.2 (0.02)		30.1 (0.01)	30.2 (0.02)	
Current smoker	17.2 (0.02)	17.0 (0.04)		15.4 (0.01)	15.3 (0.03)		15.6 (0.01)	15.5 (0.02)		16.7 (0.01)	16.7 (0.02)		16.9 (0.01)	16.8 (0.02)	
Body mass index, kg/m^2^	33.0 (7.1)	33.1 (7.1)	0.951	33.2 (6.9)	33.2 (6.9)	0.962	33.4 (7.0)	33.4 (7.0)	0.967	33.3 (6.7)	33.3 (6.7)	0.970	33.7 (6.7)	33.7 (6.7)	0.972
Systolic blood pressure, mmHg	134 (18)	133 (16)	0.166	134 (17)	133 (17)	0.437	133 (17)	133 (17)	0.429	133 (17)	133 (17)	0.333	133 (17)	132 (16)	0.238
Diastolic blood pressure, mmHg	80 (11)	80 (10)	0.180	80 (10)	80 (10)	0.663	80 (10)	80 (10)	0.708	80 (10)	81 (10)	0.769	81 (10)	80 (10)	0.579
HbA1c, mmol/mol	42.9 (4.0)	42.9 (4.0)	0.897	43.5 (3.9)	43.5 (3.9)	0.918	44.2 (4.2)	44.2 (4.2)	0.934	44.4 (4.0)	44.5 (4.0)	0.939	44.7 (3.8)	44.7 (3.8)	0.940
Total cholesterol, mmol/L	4.8 (0.8)	4.8 (0.8)	0.941	4.8 (0.9)	4.8 (0.9)	0.959	4.8 (0.9)	4.8 (0.9)	0.964	4.8 (0.9)	4.8 (0.9)	0.968	4.8 (0.9)	4.8 (0.9)	0.971
Triglyceride, mmol/L	1.6 (0.8)	1.6 (0.6)	0.398	1.8 (0.8)	1.8 (0.8)	0.515	1.7 (0.8)	1.7 (0.8)	0.672	1.8 (0.8)	1.8 (0.8)	0.597	1.7 (0.8)	1.8 (0.8)	0.980
Low‐density lipoprotein cholesterol, mmol/L	2.7 (0.7)	2.7 (0.7)	0.961	2.6 (0.7)	2.6 (0.7)	0.973	2.7 (0.7)	2.7 (0.7)	0.975	2.7 (0.8)	2.7 (0.8)	0.979	2.7 (0.8)	2.7 (0.8)	0.748
High‐density lipoprotein cholesterol, mmol/L	1.3 (0.4)	1.3 (0.4)	0.746	1.3 (0.4)	1.3 (0.4)	0.755	1.3 (0.4)	1.3 (0.4)	0.940	1.2 (0.4)	1.2 (0.4)	0.975	1.2 (0.4)	1.2 (0.3)	0.349
Estimated glomerular filtration rate < 90 mL/min/1.73 m^2^, % (SE)	36.4 (0.02)	36.2 (0.05)	0.954	33.5 (0.02)	33.7 (0.03)	0.998	34.3 (0.01)	34.3 (0.03)	0.998	38.7 (0.01)	38.8 (0.01)	0.999	38.5 (0.01)	38.5 (0.02)	0.999
Antihypertensive treatment, % (SE)	29.7 (0.03)	29.8 (0.05)	0.994	29.1 (0.02)	29.1 (0.03)	0.995	31.4 (0.02)	31.4 (0.03)	0.996	31.5 (0.02)	31.5 (0.02)	0.996	36.9 (0.02)	36.9 (0.02)	0.996
Statin treatment, % (SE)	28.7 (0.03)	28.7 (0.05)	0.994	27.5 (0.02)	27.6 (0.03)	0.995	28.4 (0.01)	28.4 (0.03)	0.996	29.4 (0.02)	29.4 (0.02)	0.996	34.3 (0.02)	34.3 (0.02)	0.996
Antiplatelet or anticoagulant treatment, % (SE)	3.1 (0.02)	3.2 (0.02)	0.998	1.5 (0.01)	1.5 (0.01)	0.999	1.0 (0.004)	1.0 (0.01)	0.999	0.8 (0.003)	0.8 (0.004)	0.999	0.9 (0.003)	0.9 (0.005)	0.999

*Note*: The continuous variables have been presented as weighted means (standard error), and the categorical variables have been presented as weighted percentage (standard error) with weights applied from the entropy matching. The landmark indicates a fixed time point after cohort entry, and those who were alive at the landmark date have been included.

Abbreviations: SE, standard error; T2D, type 2 diabetes.

Both the 5‐year and 10‐year crude and weighted incidence rates of dementia were more likely to be higher in the group with T2D onset (exposure group) across all landmark analyses. Specifically, at the 5‐year landmark, the weighted 5‐year incidence rate was 8.31 (95% confidence interval [CI]: 6.17 to 10.96) per 1000 person‐years in the exposure group, compared to 2.79 (95% CI: 1.62 to 4.47) per 1000 person‐years in the non‐exposure group. Similarly, for the 10‐year weighted incidence rate in the exposure group was 9.76 (95% CI: 4.87 to 17.46) per 1000 person‐years, while it was 3.26 (95% CI: 0.89 to 8.35) per 1000 person‐years in the non‐exposure group, as detailed in Table 2. Similarly, the 5‐year and 10‐year crude and weighted incidence rates of vascular dementia were consistently higher in the exposure group across all landmark analyses, as detailed in Table S4.

**TABLE 2:** 5‐Year and 10‐year incidence rates of dementia among cases compared between people with IGT with and without the onset of type 2 diabetes for 1‐ to 5‐year landmark analysis: Estimations from the final cohort.

	5‐Year incidence rates		10‐Year incidence rates	
Parameter	Exposure: with onset of T2D	Non‐exposure: without onset of T2D	Exposure: with onset of T2D	Non‐exposure: without onset of T2D
	Estimations from the final cohort without entropy weighting			
**1‐Year landmark analysis**	7.18 (1.96 to 18.39)	3.17 (1.52 to 5.84)	10.10 (1.22 to 36.49)	1.68 (0.94 to 2.77)
**2‐Year landmark analysis**	8.08 (2.62 to 18.85)	2.21 (1.31 to 3.49)	8.88 (4.06 to 16.85)	1.63 (1.03 to 2.45)
**3‐Year landmark analysis**	10.11 (4.62 to 19.20)	2.24 (1.43 to 3.33)	8.59 (4.29 to 15.36)	2.14 (1.47 to 3.02)
**4‐Year landmark analysis**	6.02 (2.21 to 13.10)	2.66 (1.75 to 3.87)	5.95 (2.72 to 11.29)	2.50 (1.73 to 3.49)
**5‐Year landmark analysis**	8.20 (3.30 to 16.89)	2.33 (1.36 to 3.73)	8.80 (4.92 to 14.51)	2.46 (1.61 to 3.60)
	Estimations from the final cohort with entropy weighting			
**1‐Year landmark analysis**	8.20 (2.01 to 19.66)	5.20 (2.77 to 8.90)	12.09 (8.64 to 16.46)	4.41 (2.69 to 6.80)
**2‐Year landmark analysis**	8.65 (5.84 to 12.35)	5.04 (2.98 to 7.97)	9.35 (7.29 to 11.81)	1.96 (1.20 to 3.03)
**3‐Year landmark analysis**	11.25 (8.59 to 14.49)	7.24 (5.29 to 9.66)	9.81 (7.78 to 12.21)	2.07 (1.53 to 2.75)
**4‐Year landmark analysis**	6.04 (2.79 to 14.52)	8.21 (6.05 to 10.87)	5.24 (4.26 to 6.37)	1.94 (0.63 to 4.53)
**5‐Year landmark analysis**	8.31 (6.17 to 10.96)	2.79 (1.62 to 4.47)	9.76 (4.87 to 17.46)	3.26 (0.89 to 8.35)

*Note*: Incidence rates were presented as per 1000 person‐years (95% confidence interval).

Abbreviation: IGT, impaired glucose tolerance; T2D, type 2 diabetes.

**FIGURE 1:**
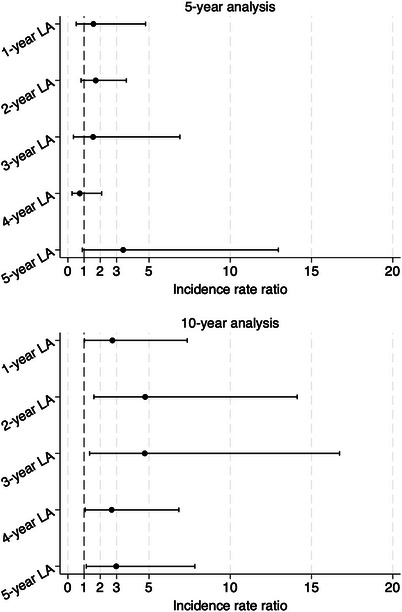
Association between the development of type 2 diabetes and the incidence rate of dementia at 5 years and 10 years in individuals with impaired glucose tolerance estimated through landmark models. LA, landmark analysis

Following the completion of step‐10 matching, the final adjusted IRR for the association between the onset of T2D and the 5‐year incidence rate of dementia did not show statistical significance at the 1‐year to 5‐year landmark analyses (refer to Figure 1 and Figure S8). However, for the 10‐year incidence rate of dementia, the final adjusted IRR demonstrated an increase from 2.74 (95% CI: 1.02 to 7.35) at the 1‐year landmark analysis to 4.76 (95% CI: 1.60 to 14.12) at the 2‐year landmark analysis, followed by a decrease to 2.98 (95% CI: 1.13 to 7.82) at the 5‐year landmark analysis (see Figure 1 and Figure S9). Similarly, the adjusted IRR for the 5‐year incidence rate of vascular dementia was not significant at the 5‐year landmark. In contrast, the adjusted IRR for the 10‐year incidence rate of vascular dementia was significant, estimated as 4.37 (95% CI: 2.88 to 6.63), as shown in Figure S10.

**FIGURE 2:**
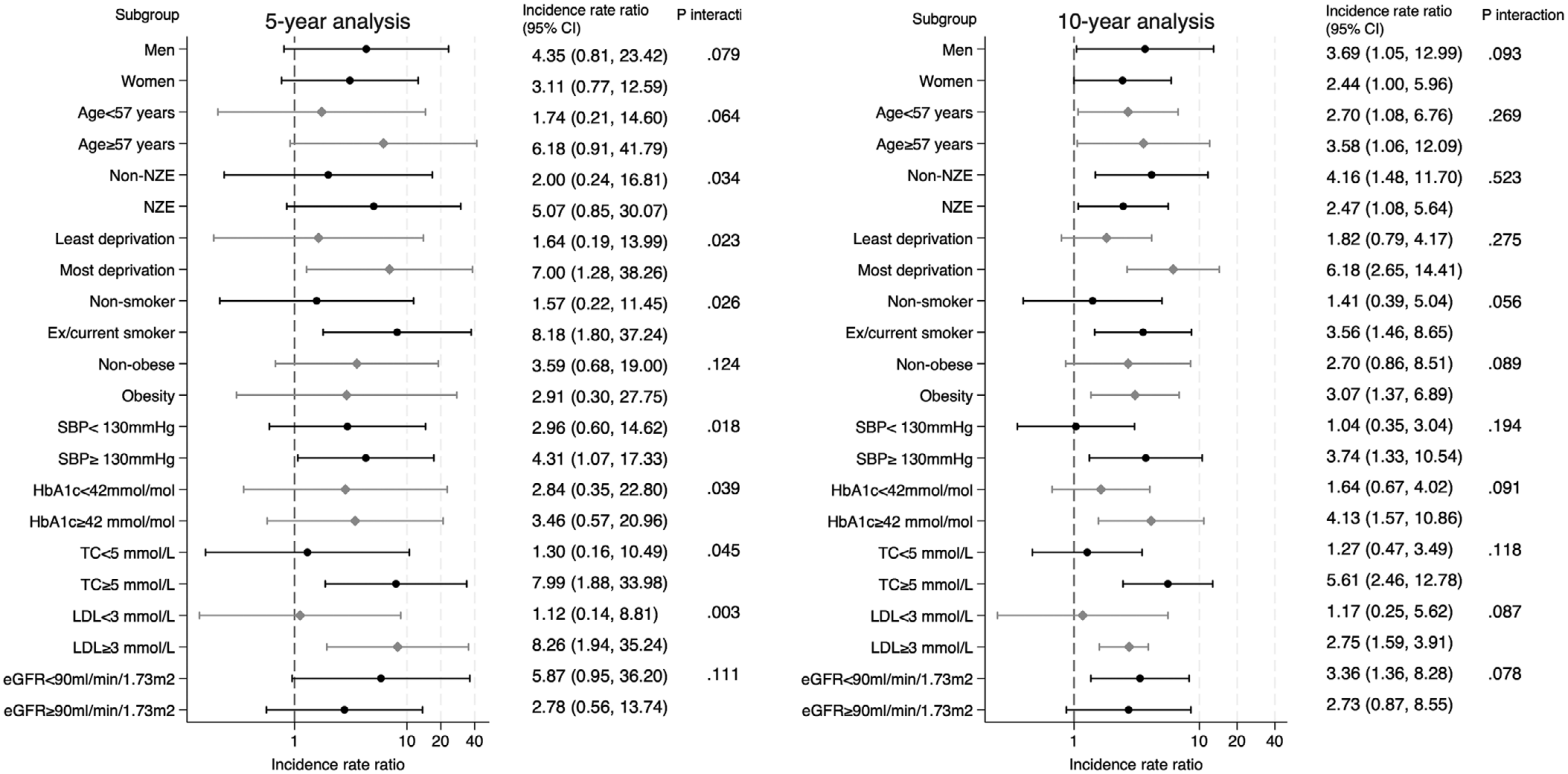
Stratified adjusted incidence rate ratio for the 5‐year and 10‐year incidence rate of dementia on the 5‐year landmark using the final tapered matched models. eGFR, estimated glomerular filtration rate; HbA1c, glycated hemoglobin; LDL, low density lipoprotein; SBP, systolic blood pressure; TC, total cholesterol.

In the 5‐year landmark analysis for subgroups, no significant association was observed between the onset of T2D and the 5‐year incidence rate of dementia in each subgroup (refer to Figure 2). However, for the 10‐year dementia incidence rate, the adjusted IRR showed a statistically significant increase in individuals with non‐NZE ethnicity, those in the most deprived group, smokers, and those with higher SBP, HbA1c, TC, and LDL (see Figure 2). The adjusted IRR did not demonstrate significant differences based on sex, age‐group, obesity status, or eGFR, as indicated by the *P*‐values for interactions (see Figure 2).

In the 5‐year landmark analysis, the adjusted IRR for the association between the onset of T2D and the 5‐year incidence rate of vascular dementia revealed a statistically significant increase among individuals with non‐NZE ethnicity, those in the most deprived groups, and those with higher SBP, HbA1c, TC, and LDL, as illustrated in Figure S10. Additionally, for the 10‐year incidence rate of vascular dementia, the adjusted IRR indicated a statistically significant increase in older individuals, those with non‐NZE ethnicity, individuals in the most deprived groups, and those with elevated SBP, HbA1c, TC, and LDL, as shown in Figure S10.

## 4 DISCUSSION

In New Zealand, our study identified a significant increase in the 10‐year dementia incidence rate among individuals with IGT who developed T2D within 1–5 years. The consistency of adjusted IRR during this period underscores the long‐term impact of T2D on cognitive decline. Subgroup analyses suggest the necessity for targeted preventive strategies, particularly for non‐NZ European ethnicities and socioeconomically deprived groups, which aligns with current literature indicating the value of lifestyle changes, smoking cessation, and diabetes screening in dementia prevention among the IGT population.^24^

While prior research has indicated a modest link between prediabetes and dementia,^2^, ^25^, ^26^ the specific impact of transitioning from IGT to type 2 diabetes T2D on dementia risk remains less explored. Our study, aligning with a recent US cohort study,^12^ confirms this significant association, showing similar adjusted IRR in individuals around 60 years at T2D onset. However, unlike the US study where age influenced dementia risk, our analysis found no significant interaction by age, suggesting differences in population profiles and hyperglycemia measures between the studies.

Our subgroup analysis further emphasizes the relationship between T2D onset and a heightened 10‐year dementia incidence, especially among IGT patients of non‐NZE ethnicity and those facing socioeconomic hardships. Notably, in NZ, the IGT prevalence ranged from 6.7% to 7.9% during 2002–2003.^27^ Evidence shows structured lifestyle changes for IGT individuals significantly curb diabetes progression,^28^ emphasizing the value of targeted prevention strategies in this demographic.

Consistent with our findings, diabetes significantly increases the risk for all‐cause and vascular dementia, as shown by meta‐analyses.^7^ This highlights the critical need for effective diabetes management to prevent cognitive decline, especially in those with IGT. Our study contributes to the evidence advocating for proactive diabetes control to lessen dementia's impact.

The elevated dementia incidence in IGT patients developing T2D may be due to shared risk factors like obesity and poor diet.^29^, ^30^ This study discounts BMI and socioeconomic status as major contributors, pointing to a need for exploring physical activity and diet's roles further. Mechanisms such as glucose toxicity,^31^, ^32^, ^33^, ^34^, ^35^, ^36^ insulin resistance,^32^, ^33^, ^36^ and microvascular dysfunction could underpin this link,^31^, ^35^ with hyperglycemia potentially affecting neuronal insulin receptors and contributing to brain pathology through oxidative and inflammatory stress.^31^, ^37^, ^38^, ^39^ These insights necessitate deeper investigation into the complex interplay between diabetes and dementia.

Our study highlights crucial clinical and public health insights, revealing that IGT individuals developing T2D face a heightened 10‐year dementia incidence rate. It underscores the need for healthcare providers to screen for dementia proactively and offer targeted counselling early on, especially within a decade of diabetes diagnosis.^40^

Preventing T2D onset in IGT individuals could reduce dementia incidence,^29^ emphasizing public health's need for targeted dementia screening, especially for high‐risk groups. Prioritizing deprived or minority groups, revealed to have higher dementia rates, underscores the importance of lifestyle interventions, such as smoking cessation and physical activity, in dementia prevention.^41^, ^42^ Collaborative efforts between healthcare providers and public health officials are vital for mitigating dementia's burden through early identification and intervention strategies.^24^

The elevated IRR identified in more socioeconomically deprived groups may underscore not only the heightened risk of dementia associated with T2D but also the potential exacerbation of this risk due to barriers in accessing effective diabetes management. This observation suggests that the severity of diabetes and the resultant risk of dementia could be significantly influenced by socioeconomic factors, highlighting the need for targeted interventions to improve diabetes care and preventive measures against dementia within these communities.

Secondary analysis on vascular dementia corroborates the pronounced 10‐year increase in incidence post‐T2D onset. This association accentuates the intersection between metabolic disorders and vascular health in dementia pathogenesis, suggesting a vascular component in T2D's influence on cognitive decline. This highlights comprehensive management strategies encompassing both glycemic and vascular health as crucial for mitigating the vascular dementia burden.^43^, ^44^

Reflecting on the difference in dementia incidence rates between the 5‐year and 10‐year periods post‐T2D onset, our findings suggest a cumulative effect of T2D that becomes more evident over time. This highlights the importance of patient demographics in understanding T2D's full impact on dementia risk and suggests that duration of T2D is a significant factor in cognitive decline.

Our analysis across varied landmark‐follow up periods cautiously avoided overinterpreting effect modification findings due to the inherent complexities of interpreting multiple models. The identified consistent patterns suggest areas for deeper exploration. However, these observations are preliminary, highlighting the need for future research with targeted designs to validate these findings and unravel the mechanisms at play.

Addressing immortal bias posed a significant challenge in our investigation into how T2D onset affects dementia incidence in IGT patients. This bias, more intricate than standard confounding, became apparent in survival comparisons between individuals with and without T2D. The issue stems from treating T2D onset as an adjusted index date, which unfairly extends the survival period for those who develop T2D, thus skewing the results. Our use of landmark analysis was instrumental in mitigating this bias, providing more reliable estimates by evaluating T2D onset and dementia incidence at predetermined times. Chosen for its clarity, the landmark analysis method offers advantages over time‐varying analyses, which, despite providing a comprehensive view of incidence over time, introduce additional modelling complexity. The landmark approach's simplicity is particularly beneficial for its direct relevance to clinical practice and policy formulation. Nonetheless, future studies exploring time‐varying analyses could yield further insights into the nuanced effects of T2D on dementia risk.

Our study showcases notable strengths. First, it is a significant multi‐ethnic cohort study in New Zealand, exploring the T2D onset and dementia incidence rates over 5‐ and 10‐year periods, making it one of the most extensive of its kind globally. The cohorts, encompassing patients from various general practices, were linked to large databases for effective follow‐up and accurate dementia case recording, verified by ICD codes. Second, the use of landmark analysis and tapered matching techniques provided a robust method to mitigate immortal bias, enabling a detailed evaluation of dementia incidence rates and the impact of confounders, akin to a quasi‐trial comparison between IGT patients with and without T2D onset.

Despite these numerous strengths, some limitations exist in our study. One limitation is the lack of full national representation of the sample and the participating general practices in New Zealand, which may affect the generalizability of the findings to the broader population. Additionally, certain risk factors for dementia, such as dietary information, physical activity levels, and genetic variants, were not accessible in the available data. Future studies should consider incorporating these risk factors into their analyses to enhance our understanding of the relationship between IGT, T2D, and dementia. While our study identifies T2D as a risk factor for dementia in individuals with IGT, further research explicitly examining mediation effects would be valuable to fully understand this relationship. The dataset used in this study provided measurements of lifestyle factors and clinical measurements by index date without subsequent repeated measures. Therefore, the analysis was limited to the information available by the index date. Future studies with access to longitudinal data would be valuable to assess how changes in lifestyle factors and clinical measurements over time may influence the risk of dementia. While we recognize the extensive scope of our data linkage, and the concerted efforts by healthcare providers to undertake regular healthcare screening among DCSS patients, we acknowledge the possibility that a fraction of patients may not have engaged with any healthcare services. To deepen our understanding of this potential influence, future research utilizing national screening data could provide valuable insights into the associations identified in our study.

Regarding the selection of individuals with IGT using DCSS data and their dependency on an OGTT, it is important to note the targeted nature of the DCSS dataset. The dataset primarily encompasses individuals under the care of general practices involved in the DCSS, which not only manage existing diabetes patients but also actively screen at‐risk populations based on weight, ethnicity, family history, and age criteria. This screening process includes both fasting blood glucose tests and, where indicated, OGTTs to accurately identify individuals with IGT. While our methodology ensures that most individuals eligible for OGTT screening based on their risk profile are tested, we recognize the possibility, that some at‐risk individuals might not participate in screening even when invited. This scenario, though likely representing a small fraction of the target population, could potentially introduce a degree of selection bias into our study. It is a limitation that, while slight, underscores the importance of continuous efforts to enhance screening uptake among high‐risk groups. Future research may benefit from exploring strategies to increase screening participation, thereby reducing the risk of selection bias and further bolstering the internal and external validity of findings in similar studies.

In summary, our study provides new insights into how T2D onset in IGT individuals elevates dementia rates, stressing the need for early intervention and lifestyle changes. This emphasizes the necessity of lifestyle improvements and diabetes prevention to lower dementia risks. Future research should validate these findings and explore the mechanisms involved.

## AUTHOR CONTRIBUTIONS

Dahai Yu, Zhanzheng Zhao, and David Simmons contributed to the design of the study and the conduct during all its phases. Dahai Yu, Zheng Wang, Bingjie Qu, Yamei Cai, Zhanzheng Zhao, and David Simmons contributed to the processing and the statistical handling of the data to generate the results. Dahai Yu, Zheng Wang, Yamei Cai, Bingjie Qu, Uchechukwu Levi Osuagwu, Karen Pickering, John Baker, Richard Cutfield, Brandon J. Orr‐Walker, Gerhard Sundborn, Zhanzheng Zhao, and David Simmons contributed to the discussions on the interpretation of the results and their appropriate presentation. David Simmons contributed to the patient and public input at all stages of the study. Dahai Yu, Zheng Wang, Yamei Cai, Uchechukwu Levi Osuagwu, Karen Pickering, John Baker, Richard Cutfield, Bingjie Qu, Brandon J. Orr‐Walker, Gerhard Sundborn, Zhanzheng Zhao, and David Simmons commented on the draft of the final manuscript. David Simmons initiated the project and secured the funding. Dahai Yu, Zheng Wang, Yamei Cai, Uchechukwu Levi Osuagwu, Karen Pickering, John Baker, Richard Cutfield, Bingjie Qu, Brandon J. Orr‐Walker, Gerhard Sundborn, Zhanzheng Zhao, and David Simmons produced the first draft of the manuscript.

## CONFLICT OF INTEREST STATEMENT

All authors declare no conflict of interest. Author disclosures are available in the supporting information.

## CONSENT FOR PUBLICATION

All authors have provided consent for publication of the manuscript.

## Supporting information

Supporting Information

Supporting Information

## Data Availability

The datasets analyzed in the current study are not publicly available because of agreements with the primary care organizations and Ministry of Health who provided the data.
