# Supplementary material for: Long‐term impact of type 2 diabetes onset on dementia incidence rate among New Zealanders with impaired glucose tolerance: A tapered‐matched landmark analysis over 25 years
Source: Alzheimers Dement. 2024 Jun 14;20(7):4423–33. doi: 10.1002/alz.13855 (PMC11247710; doi:10.1002/alz.13855)

**Table-S1**. ICD-9 and ICD-10 codes and terms used to identify dementia

| **Code Type** | **Code** | **Term** |
| --- | --- | --- |
| ICD 9 Code | 290.2 | Senile dementia, depressed or paranoid type |
| ICD 9 Code | 290.3 | Senile dementia with acute confusional state |
| ICD 9 Code | 290.4 | Arteriosclerotic dementia |
| ICD 9 Code | 291.2 | Other alcoholic dementia |
| ICD 9 Code | 294.1 | Dementia in other conditions classified elsewhere |
| ICD 9 Code | 331 | Alzheimer's disease |
| ICD 9 Code | 331.1 | Pick's disease |
| ICD 9 Code | 331.2 | Senile degeneration of brain |
| ICD 9 Code | 331.5 | Creutzfeldt-Jakob disease |
| ICD 10 Code | A81.0 | Sporadic Creutzfeldt-Jakob disease |
| ICD 10 Code | F00 | Dementia in Alzheimer's disease |
| ICD 10 Code | F00.0 | Dementia in Alzheimer's disease with early onset |
| ICD 10 Code | F00.1 | Dementia in Alzheimer's disease with late onset |
| ICD 10 Code | F00.2 | Dementia in Alzheimer's disease, atypical or mixed type |
| ICD 10 Code | F00.9 | Dementia in Alzheimer's disease, unspecified |
| ICD 10 Code | F01 | Vascular dementia |
| ICD 10 Code | F01.0 | Vascular dementia of acute onset |
| ICD 10 Code | F01.1 | Multi-infarct dementia |
| ICD 10 Code | F01.2 | Subcortical vascular dementia |
| ICD 10 Code | F01.3 | Mixed cortical and sub-cortical vascular dementia |
| ICD 10 Code | F01.8 | Other vascular dementia |
| ICD 10 Code | F01.9 | Vascular dementia, unspecified |
| ICD 10 Code | F02 | Dementia in other diseases classified elsewhere |
| ICD 10 Code | F02.0 | Dementia in Pick's disease |
| ICD 10 Code | F02.1 | Dementia in Creutzfeldt-Jakob disease |
| ICD 10 Code | F02.2 | Dementia in Huntington’s disease |
| ICD 10 Code | F02.3 | Dementia in Parkinson’s disease |
| ICD 10 Code | F02.4 | Dementia in HIV disease |
| ICD 10 Code | F02.8 | Dementia in other specified diseases classified elsewhere |
| ICD 10 Code | F03 | Unspecified dementia |
| ICD 10 Code | F05.1 | Delirium superimposed on dementia |
| ICD 10 Code | F10.6 | Mental and behavioural disorders due to use of alcohol - amnesic syndrome |
| ICD 10 Code | G30 | Alzheimer’s disease |
| ICD 10 Code | G30.0 | Alzheimer’s disease with early onset |
| ICD 10 Code | G30.1 | Alzheimer’s disease with late onset |
| ICD 10 Code | G30.8 | Other Alzheimer's disease |
| ICD 10 Code | G30.9 | Alzheimer's disease unspecified |
| ICD 10 Code | G31.0 | Circumscribed brain atrophy |
| ICD 10 Code | G31.1 | Senile degeneration of brain |
| ICD 10 Code | G31.8 | Other specified degenerative diseases of nervous system |
| ICD 10 Code | I67.3 | Binswanger's disease |

**Table-S2.** Calculation of absolute incidence rates from Poisson regression models

| In our analysis, we aimed to estimate the absolute incidence rates of the outcome for different exposure groups. After fitting a Poisson regression model, we utilised the margins command in Stata to calculate the adjusted incidence rates, as follows:  *poisson outcome i.exposure , irr exp(person_time)*  *margins exposure, predict(ir)*  Where:  *outcome* is the number of events,  exposure is the indicator for the exposure group,  *person_time* is the offset for the model representing the person-time at risk. |
| --- |

**Table-S3**. Comparison of patients with and without onset of type 2 diabetes in patients with impaired glucose tolerance in the unmatched and coarsened exact matching cohorts

|  | **1-year landmark** | | | **2-year landmark** | | | **3-year landmark** | | | **4-year landmark** | | | **5-year landmark** | | |
| --- | --- | --- | --- | --- | --- | --- | --- | --- | --- | --- | --- | --- | --- | --- | --- |
|  | Without T2D onset | With T2D onset | *P*-value | Without T2D onset | With T2D onset | *P*-value | Without T2D onset | With T2D onset | *P*-value | Without T2D onset | With T2D onset | *P*-value | Without T2D onset | With T2D onset | *P*-value |
|  | N=24,664 | N=180 |  | N=24519 | N=379 |  | N=22614 | N=587 |  | N=19,489 | N=744 |  | N=15,346 | N=837 |  |
|  | **Unmatched** | | | | | | | | | | | | | | |
| Age, years | 55.7 (12.9) | 57.3 (13.3) |  | 55.7 (12.9) | 57.7 (12.4) | 0.004 | 55.4 (12.9) | 56.3 (13.4) | 0.127 | 55.5 (12.7) | 56.2 (13.0) | 0.168 | 55.9 (12.6) | 56.4 (12.7) | 0.343 |
| Female Gender, n (%) | 13256 (53.8) | 99 (55.0) |  | 13180 (53.8) | 200 (52.8) | 0.703 | 12153 (53.7) | 316 (53.8) | 0.965 | 10459 (53.7) | 405 (54.4) | 0.680 | 8186 (53.3) | 444 (53.1) | 0.867 |
| New Zealand European, n (%) | 12329 (50.0) | 83 (46.1) |  | 12224 (49.9) | 177 (46.7) | 0.223 | 10856 (48.0) | 254 (43.3) | 0.023 | 9525 (48.9) | 316 (42.5) | 0.001 | 7924 (51.6) | 366 (43.7) | <0.0001 |
| Enrol cohort, n (%) |  |  |  |  |  |  |  |  |  |  |  |  |  |  |  |
| 1994-1998 | 125 (0.5) | 2 (1.1) |  | 123 (0.5) | 10 (2.6) | <0.0001 | 119 (0.5) | 12 (2.0) | <0.0001 | 117 (0.6) | 13 (1.8) | <0.0001 | 114 (0.7) | 18 (2.2) | <0.0001 |
| 1999-2003 | 543 (2.2) | 12 (6.7) |  | 535 (2.2) | 25 (6.6) |  | 525 (2.3) | 48 (8.2) |  | 514 (2.6) | 67 (9.0) |  | 504 (3.3) | 87 (10.4) |  |
| 2004-2008 | 1831 (7.4) | 35 (19.4) |  | 1808 (7.4) | 65 (17.2) |  | 1782 (7.9) | 87 (14.8) |  | 1752 (9.0) | 110 (14.8) |  | 1724 (11.2) | 158 (18.9) |  |
| 2009-2013 | 9022 (36.6) | 69 (38.3) |  | 8942 (36.5) | 140 (36.9) |  | 8876 (39.3) | 221 (37.7) |  | 8831 (45.3) | 332 (44.6) |  | 8805 (57.4) | 439 (52.5) |  |
| 2014-2018 | 13143 (53.3) | 62 (34.4) |  | 13111 (53.5) | 139 (36.7) |  | 11312 (50.0) | 219 (37.3) |  | 8275 (42.5) | 222 (29.8) |  | 4199 (27.4) | 135 (16.1) |  |
| IMD group (NZDep13 scale) |  |  |  |  |  |  |  |  |  |  |  |  |  |  |  |
| Least Deprivation: IMD-1 (1 or 2) | 3611 (14.6) | 18 (10.0) |  | 3597 (14.7) | 38 (10.0) | 0.108 | 3265 (14.4) | 59 (10.1) | 0.003 | 2903 (14.9) | 75 (10.1) | <0.0001 | 2187 (14.3) | 75 (9.0) | <0.0001 |
| IMD-2 (3 or 4) | 4343 (17.6) | 20 (11.1) |  | 4318 (17.6) | 54 (14.3) |  | 3813 (16.9) | 83 (14.1) |  | 3342 (17.2) | 108 (14.5) |  | 2673 (17.4) | 126 (15.1) |  |
| IMD-3 (5 or 6) | 3108 (12.6) | 23 (12.8) |  | 3087 (12.6) | 49 (12.9) |  | 2795 (12.4) | 72 (12.3) |  | 2332 (12.0) | 93 (12.5) |  | 1954 (12.7) | 112 (13.4) |  |
| IMD-4 (7 or 8) | 3566 (14.5) | 32 (17.8) |  | 3541 (14.4) | 62 (16.4) |  | 3345 (14.8) | 90 (15.3) |  | 2972 (15.3) | 114 (15.3) |  | 2440 (15.9) | 144 (17.2) |  |
| Most Deprivation: IMD-5 (9 or 10) | 10036 (40.7) | 87 (48.3) |  | 9976 (40.7) | 176 (46.4) |  | 9396 (41.6) | 283 (48.2) |  | 7940 (40.7) | 354 (47.6) |  | 6092 (39.7) | 380 (45.4) |  |
| Smoking status, n (%) |  |  |  |  |  |  |  |  |  |  |  |  |  |  |  |
| Never smoking | 14665 (59.5) | 98 (54.4) |  | 14592 (59.5) | 200 (52.8) | 0.028 | 13458 (59.5) | 319 (54.3) | 0.038 | 11649 (59.8) | 410 (55.1) | 0.036 | 9230 (60.2) | 466 (55.7) | 0.025 |
| Ex-smoker | 6543 (25.5) | 53 (29.4) |  | 6495 (26.5) | 119 (31.4) |  | 5920 (26.2) | 170 (29.0) |  | 5079 (26.1) | 213 (28.6) |  | 3991 (26.0) | 234 (28.0) |  |
| Current Smoker | 3456 (14.0) | 29 (16.1) |  | 3432 (14.0) | 60 (15.8) |  | 3236 (14.3) | 98 (16.7) |  | 2761 (14.2) | 121 (16.3) |  | 2125 (13.9) | 137 (16.4) |  |
| Body mass index, kg/m^2^ | 31.2 (6.4) | 33.6 (7.3) |  | 31.2 (6.4) | 33.6 (6.9) | <0.0001 | 31.3 (13.4) | 33.8 (7.0) | <0.0001 | 31.2 (6.3) | 33.6 (6.7) | <0.0001 | 31.1 (6.2) | 33.7 (6.7) | <0.0001 |
| Systolic blood pressure, mmHg | 131 (16) | 132 (17) |  | 131 (16) | 133 (17) | 0.015 | 131 (16) | 133 (17) | 0.006 | 131 (16) | 134 (17) | <0.0001 | 131 (16) | 134 (18) | <0.0001 |
| Diastolic blood pressure, mmHg | 79 (10) | 79 (10) |  | 79 (10) | 80 (11) | 0.019 | 79 (10) | 81 (10) | 0.003 | 80 (10) | 81 (10) | <0.0001 | 80 (10) | 81 (11) | 0.001 |
| HbA1c, mmol/mol | 42.1 (3.2) | 42.9 (3.9) |  | 42.1 (3.2) | 43.5 (3.9) | <0.0001 | 42.3 (3.2) | 44.0 (4.1) | <0.0001 | 42.4 (3.1) | 44.4 (4.0) | <0.0001 | 42.6 (3.1) | 44.5 (4.0) | <0.0001 |
| Total cholesterol, mmol/L | 5.1 (1.0) | 4.7 (1.0) |  | 5.1 (1.0) | 4.8 (1.0) | <0.0001 | 5.1 (1.0) | 4.8 (1.0) | <0.0001 | 5.1 (1.0) | 4.9 (1.0) | <0.0001 | 5.1 (1.0) | 4.9 (1.0) | <0.0001 |
| Triglyceride, mmol/L | 1.7 (0.8) | 1.6 (0.7) |  | 1.7 (0.8) | 1.7 (0.8) | 0.170 | 1.7 (0.8) | 1.8 (0.9) | 0.004 | 1.7 (0.8) | 1.8 (0.9) | <0.0001 | 1.6 (0.7) | 1.7 (0.8) | <0.0001 |
| Low-density lipoprotein cholesterol, mmol/L | 2.8 (0.7) | 2.6 (0.7) |  | 2.8 (0.7) | 2.6 (0.7) | <0.0001 | 2.8 (0.7) | 2.7 (0.7) | <0.0001 | 2.8 (0.7) | 2.7 (0.8) | <0.0001 | 2.8 (0.7) | 2.7 (0.8) | <0.0001 |
| High-density lipoprotein cholesterol, mmol/L | 1.3 (0.4) | 1.3 (0.3) |  | 1.3 (0.4) | 1.3 (0.3) | 0.154 | 1.3 (0.4) | 1.3 (0.3) | 0.002 | 1.3 (0.4) | 1.2 (0.3) | <0.0001 | 1.3 (0.4) | 1.2 (0.3) | <0.0001 |
| estimated Glomerular filtration rate<90 ml/min/1.73 m^2^ | 6543 (26.5) | 53 (29.4) |  | 7771 (31.7) | 124 32.7) | <0.0001 | 7403 (32.7) | 203 (34.6) | 0.003 | 6711 (34.4) | 277 (37.2) | 0.001 | 4530 (29.5) | 294 (35.1) | <0.0001 |
| Antihypertensive treatment, n (%) | 2499 (10.1) | 46 (25.6) |  | 2479 (10.1) | 102 (26.9) | <0.0001 | 2431 (10.8) | 163 (27.8) | <0.0001 | 2356 (12.1) | 222 (29.8) | <0.0001 | 2263 (14.8) | 301 (36.0) | <0.0001 |
| Statin treatment, n (%) | 2051 (8.3) | 43 (23.9) |  | 2037 (8.3) | 90 (23.8) | <0.0001 | 2001 (8.9) | 142 (24.2) | <0.0001 | 1947 (10.0) | 194 (26.1) | <0.0001 | 1877 (12.2) | 265 (31.7) | <0.0001 |
| Antiplatelet or anticoagulant treatment, n (%) | 74 (0.3) | 5 (2.8) |  | 73 (0.3) | 6 (1.6) | <0.0001 | 72 (0.3) | 10 (1.7) | <0.0001 | 68 (0.4) | 12 (1.6) | <0.0001 | 64 (0.4) | 15 (1.8) | <0.0001 |
|  | **CEM** | | | | | | | | | | | | | | |
| N | 2000 | 157 |  | 3925 | 329 |  | 5156 | 505 |  | 5308 | 631 |  | 4556 | 691 |  |
| Age, years | 54.1 (13.8) | 57.6 (13.3) |  | 54.5 (13.5) | 57.9 (12.5) | <0.0001 | 53.6 (13.3) | 56.3 (13.5) | <0.0001 | 54.1 (13.2) | 56.2 (13.1) | <0.0001 | 53.8 (12.6) | 56.1 (13.0) | <0.0001 |
| Female Gender, n (%) | 1222 (61.0) | 86 (54.8) |  | 2044 (52.1) | 171 (52.0) | 0.972 | 2739 (53.1) | 265 (52.5) | 0.781 | 2933 (55.3) | 337 (53.4) | 0.377 | 2421 (53.1) | 362 (52.4) | 0.712 |
| New Zealand European, n (%) | 597 (29.9) | 69 (44.0) |  | 1309 (33.4) | 150 (45.6) | <0.0001 | 1610 (31.2) | 213 (42.2) | <0.0001 | 1872 (35.3) | 267 (42.3) | <0.0001 | 1604 (35.2) | 300 (43.4) | <0.0001 |
| Enrol cohort, n (%) |  |  |  |  |  |  |  |  |  |  |  |  |  |  |  |
| 1994-1998 | 5 (0.3) | 2 (1.3) |  | 12 (0.3) | 10 (3.0) | <0.0001 | 13 (0.25) | 11 (2.2) | <0.0001 | 16 (0.3) | 10 (1.6) | <0.0001 | 18 (0.4) | 15 (2.2) | <0.0001 |
| 1999-2003 | 42 (2.1) | 7 (4.5) |  | 81 (2.1) | 17 (5.2) |  | 114 (2.2) | 39 (7.7) |  | 140 (2.6) | 56 (8.9) |  | 130 (2.9) | 68 (9.8) |  |
| 2004-2008 | 104 (5.2) | 28 (17.8) |  | 229 (5.8) | 51 (15.5) |  | 303 (5.9) | 65 (12.9) |  | 401 (7.6) | 82 (13.0) |  | 413 (9.1) | 114 (16.5) |  |
| 2009-2013 | 923 (46.2) | 61 (38.9) |  | 1679 (42.8) | 123 (37.4) |  | 2284 (44.3) | 193 (38.2) |  | 2901 (54.7) | 287 (45.5) |  | 3362 (73.8) | 380 (55.0) |  |
| 2014-2018 | 926 (46.3) | 59 (37.6) |  | 1924 (49.0) | 128 (38.9) |  | 2442 (47.4) | 197 (39.0) |  | 1850 (34.9) | 196 (31.1) |  | 633 (13.9) | 114 (16.5) |  |
| IMD group (NZDep13 scale) |  |  |  |  |  |  |  |  |  |  |  |  |  |  |  |
| Least Deprivation: IMD-1 (1 or 2) | 144 (7.2) | 17 (10.8) |  | 333 (8.5) | 32 (9.7) | <0.0001 | 431 (8.4) | 50 (9.9) | <0.0001 | 549 (10.3) | 64 (10.1) | 0.138 | 470 (10.3) | 62 (9.0) | 0.033 |
| IMD-2 (3 or 4) | 193 (9.7) | 18 (11.5) |  | 407 (10.4) | 50 (15.2) |  | 551 (10.7) | 73 (14.5) |  | 700 (13.2) | 93 (14.7) |  | 683 (15.0) | 105 (15.2) |  |
| IMD-3 (5 or 6) | 143 (7.2) | 18 (11.5) |  | 300 (7.6) | 39 (11.9) |  | 389 (7.5) | 58 (11.5) |  | 482 (9.1) | 74 (11.7) |  | 461 (10.1) | 89 (12.9) |  |
| IMD-4 (7 or 8) | 308 (15.4) | 29 (18.5) |  | 605 (15.4) | 53 (16.1) |  | 821 (15.9) | 76 (15.1) |  | 810 (15.3) | 96 (15.2) |  | 670 (14.7) | 120 (17.4) |  |
| Most Deprivation: IMD-5 (9 or 10) | 1212 (60.6) | 75 (47.8) |  | 2289 (58.1) | 155 (47.1) |  | 2964 (57.5) | 248 (49.1) |  | 2767 (52.1) | 304 (48.2) |  | 2772 (49.9) | 315 (45.6) |  |
| Smoking status, n (%) |  |  |  |  |  |  |  |  |  |  |  |  |  |  |  |
| Never smoking | 1084 (54.2) | 82 (52.2) |  | 2042 (52.0) | 174 (52.9) | 0.698 | 2879 (55.8) | 277 (54.9) | 0.817 | 3163 (59.6) | 353 (55.9) | 0.189 | 2706 (59.4) | 383 (55.4) | 0.139 |
| Ex-smoker | 562 (28.1) | 48 (30.6) |  | 1166 (29.7) | 101 (30.7) |  | 1382 (26.8) | 142 (28.1) |  | 1312 (24.7) | 174 (27.6) |  | 1117 (24.5) | 188 (27.2) |  |
| Current Smoker | 354 (17.7) | 27 (17.2) |  | 717 (18.3) | 54 (16.4) |  | 895 (17.4) | 86 (17.0) |  | 833 (15.7) | 104 (16.5) |  | 733 (16.1) | 120 (17.4) |  |
| Body mass index, kg/m^2^ | 33.8 (6.6) | 33.6 (7.3) |  | 33.5 (6.5) | 33.5 (6.9) | 0.946 | 33.2 (6.5) | 33.7 (7.0) | 0.153 | 33.0 (6.4) | 33.5 (6.7) | 0.080 | 32.7 (6.5) | 33.8 (6.7) | <0.0001 |
| Systolic blood pressure, mmHg | 132 (17) | 132 (16) |  | 134 (17) | 133 (17) | 0.563 | 133 (17) | 133 (17) | 0.775 | 133 (16) | 134 (17) | 0.079 | 132 (16) | 134 (17) | 0.016 |
| Diastolic blood pressure, mmHg | 80 (10) | 79 (10) |  | 81 (10) | 80 (11) | 0.357 | 81 (10) | 81 (10) | 0.570 | 81 (10) | 82 (10) | 0.365 | 81 (10) | 81 (11) | 0.609 |
| HbA1c, mmol/mol | 43.2 (2.8) | 43.1 (3.8) |  | 43.2 (2.8) | 43.7 (3.8) | 0.014 | 43.3 (2.8) | 44.2 (4.0) | <0.0001 | 43.4 (2.8) | 44.5 (3.9) | <0.0001 | 43.4 (2.8) | 44.8 (3.8) | <0.0001 |
| Total cholesterol, mmol/L | 5.1 (0.9) | 4.8 (1.0) |  | 5.2 (0.9) | 4.8 (1.0) | <0.0001 | 5.1 (0.9) | 4.9 (1.0) | <0.0001 | 5.2 (0.9) | 4.9 (1.0) | <0.0001 | 5.2 (0.9) | 4.9 (1.0) | <0.0001 |
| Triglyceride, mmol/L | 1.7 (0.8) | 1.7 (0.7) |  | 1.8 (0.8) | 1.8 (0.8) | 0.764 | 1.8 (0.8) | 1.8 (0.8) | 0.202 | 1.7 (0.8) | 1.8 (0.8) | 0.055 | 1.7 (0.8) | 1.8 (0.8) | 0.027 |
| Low-density lipoprotein cholesterol, mmol/L | 2.9 (0.7) | 2.7 (0.7) |  | 2.9 (0.7) | 2.6 (0.7) | <0.0001 | 2.9 (0.7) | 2.7 (0.8) | <0.0001 | 2.9 (0.7) | 2.7 (0.8) | <0.0001 | 2.9 (0.7) | 2.7 (0.7) | <0.0001 |
| High-density lipoprotein cholesterol, mmol/L | 1.3 (0.3) | 1.3 (0.3) |  | 1.3 (0.4) | 1.3 (0.3) | 0.588 | 1.3 (0.4) | 1.3 (0.3) | 0.765 | 1.3 (0.4) | 1.2 (0.3) | 0.129 | 1.3 (0.4) | 1.2 (0.3) | 0.006 |
| estimated Glomerular filtration rate<90 ml/min/1.73 m^2^ | 697 (34.9) | 50 (31.9) |  | 1417 (36.1) | 105 (31.9) | 0.077 | 1907 (37.0) | 184 (36.5) | 0.246 | 1963 (37.0) | 231 (36.6) | 0.048 | 1335 (29.3) | 227 (32.9) | <0.0001 |
| Antihypertensive treatment, n (%) | 141 (7.1) | 35 (22.3) |  | 343 (8.7) | 75 (22.8) | <0.0001 | 492 (9.5) | 123 (24.4) | <0.0001 | 614 (11.6) | 164 (26.0) | <0.0001 | 650 (14.3) | 222 (32.1) | <0.0001 |
| Statin treatment, n (%) | 116 (5.8) | 34 (21.7) |  | 271 (6.9) | 71 (21.6) | <0.0001 | 395 (7.7) | 109 (21.6) | <0.0001 | 489 (9.2) | 147 (23.3) | <0.0001 | 538 (11.8) | 200 (28.9) | <0.0001 |
| Antiplatelet or anticoagulant treatment, n (%) | 5 (3.2) | 11 (0.6) |  | 14 (0.4) | 6 (1.8) | <0.0001 | 18 (0.4) | 7 (1.4) | 0.001 | 21 (0.4) | 8 (1.3) | 0.003 | 15 (0.3) | 10 (1.5) | <0.0001 |

**Table-S4**. 5-year and 10-year incidence rates of vascular dementia among cases compared between people with IGT with and without the onset of type 2 diabetes for 1-5 year landmark analysis: estimations from the final cohort

*Incidence rates were presented as per 1,000 person-years (95% confidence interval).*

|  | **5-year incidence rates** | | **10-year incidence rates** | |
| --- | --- | --- | --- | --- |
|  | **Exposure: with onset of T2D** | **Non-exposure: without onset of T2D** | **Exposure: with onset of T2D** | **Non-exposure: without onset of T2D** |
|  | Estimations from the final cohort without entropy weighting | | | |
| **1-year landmark analysis** | 4.65 (0.12 to 25.91) | 0.61 (0.41 to 0.89) | 3.17 (0.38 to 11.47) | 0.40 (0.28 to 0.54) |
| **2-year landmark analysis** | 1.45 (0.04 to 8.06) | 0.46 (0.29 to 0.69) | 3.40 (0.93 to 8.72) | 0.42 (0.29 to 0.58) |
| **3-year landmark analysis** | 1.03 (0.03 to 5.71) | 0.44 (0.26 to 0.68) | 1.95 (0.40 to 5.70) | 0.47 (0.32 to 0.67) |
| **4-year landmark analysis** | 1.74 (0.21 to 6.28) | 0.51 (0.30 to 0.81) | 1.62 (0.33 to 4.73) | 0.57 (0.38 to 8.32) |
| **5-year landmark analysis** | 4.96 (1.61 to 11.56) | 0.73 (0.41 to 1.21) | 4.10 (1.87 to 7.78) | 0.65 (0.41 to 0.98) |
|  | Estimations from the final cohort with entropy weighting | | | |
| **1-year landmark analysis** | 4.16 (1.35 to 9.76) | 0.48 (0.01 to 2.67) | 4.24 (0.10 to 23.60 | 0.32 (0.16 to 0.55) |
| **2-year landmark analysis** | 3.24 (0.08 to 18.04) | 0.25 (0.11 to 0.50) | 2.29 (0.48 to 3.27) | 0.11 (0.05 to 0.22) |
| **3-year landmark analysis** | 1.84 (0.88 to 3.38) | 0.23 (0.08 to 0.50) | 3.60 (0.74 to 10.51) | 0.17 (0.06 to 0.36) |
| **4-year landmark analysis** | 2.99 (0.36 to 10.78) | 0.76 (0.58 to 0.99) | 4.92 (1.01 to 14.37) | 0.52 (0.19 to 1.13) |
| **5-year landmark analysis** | 3.27 (0.40 to 11.82) | 0.82 (0.51 to 1.22) | 4.31 (1.58 to 9.39) | 0.99 (0.43 to 1.94) |

**Figure S1**. Graphical representation of landmark analysis.


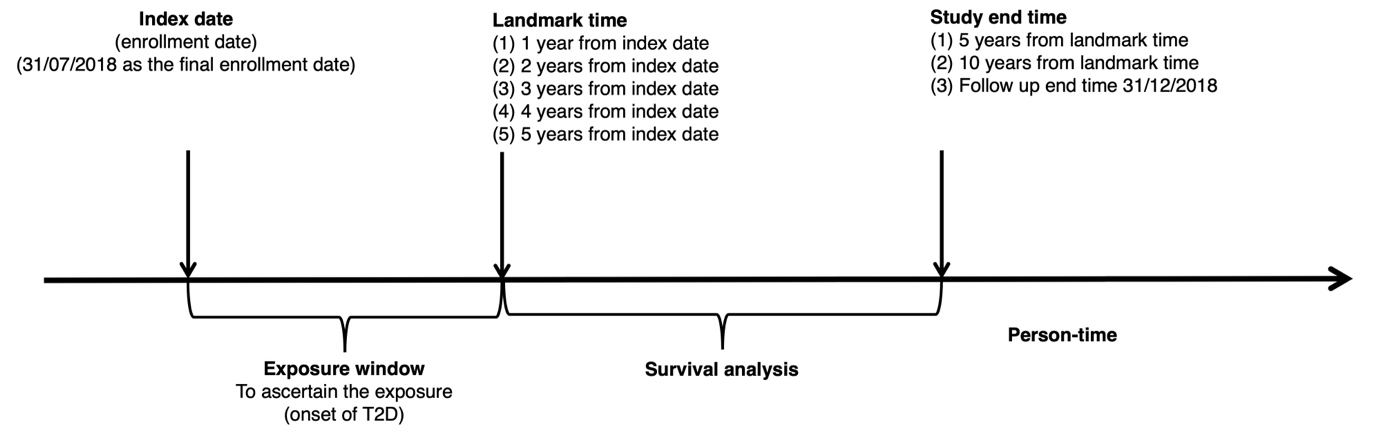


**Figure S2**. Workflow charts for matching process (1-year landmark analysis)


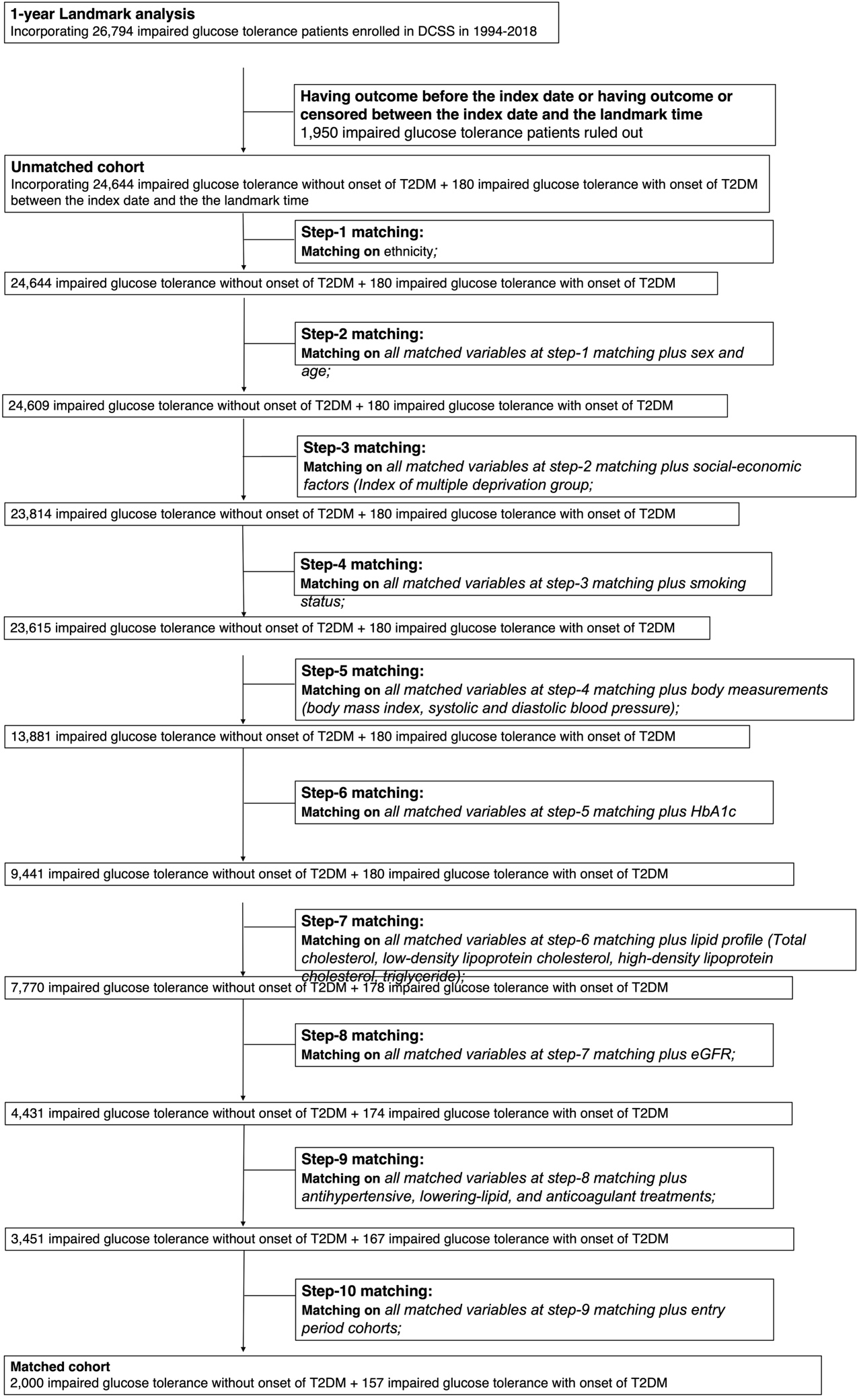


**Figure S3**. Workflow charts for matching process (2-year landmark analysis)

**
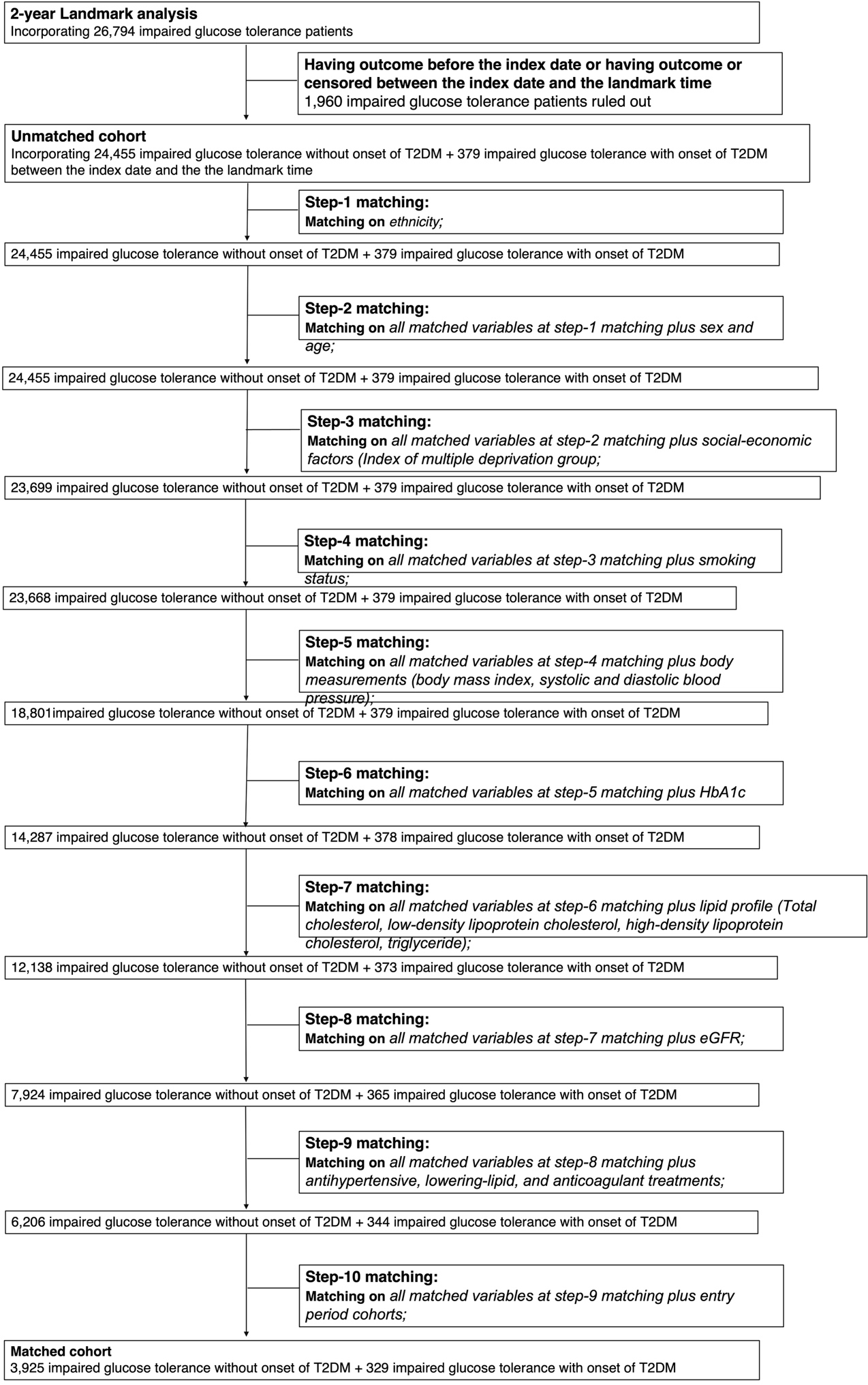
**

**Figure S4**. Workflow charts for matching process (3-year landmark analysis)


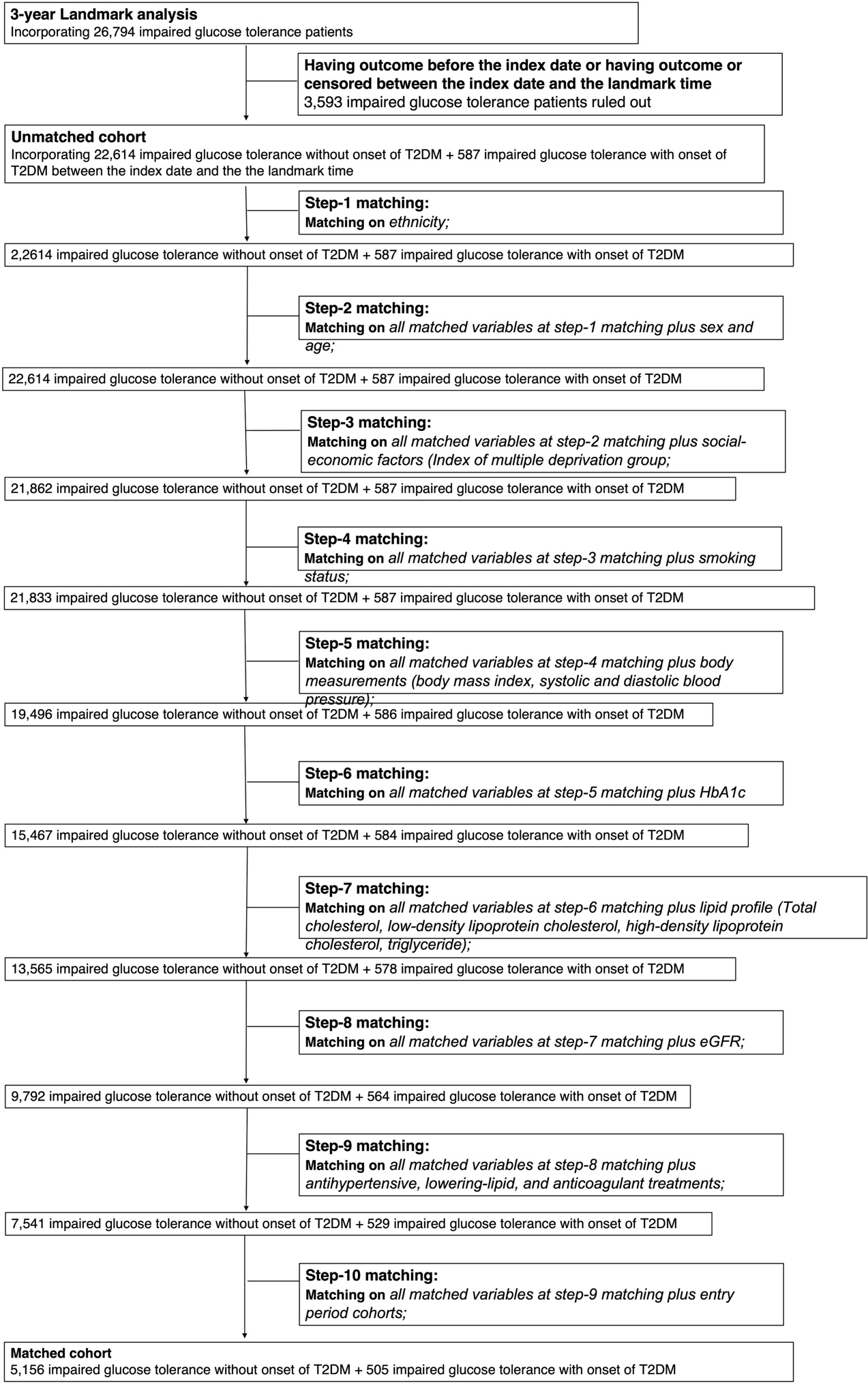


**Figure S5**. Workflow charts for matching process (4-year landmark analysis)


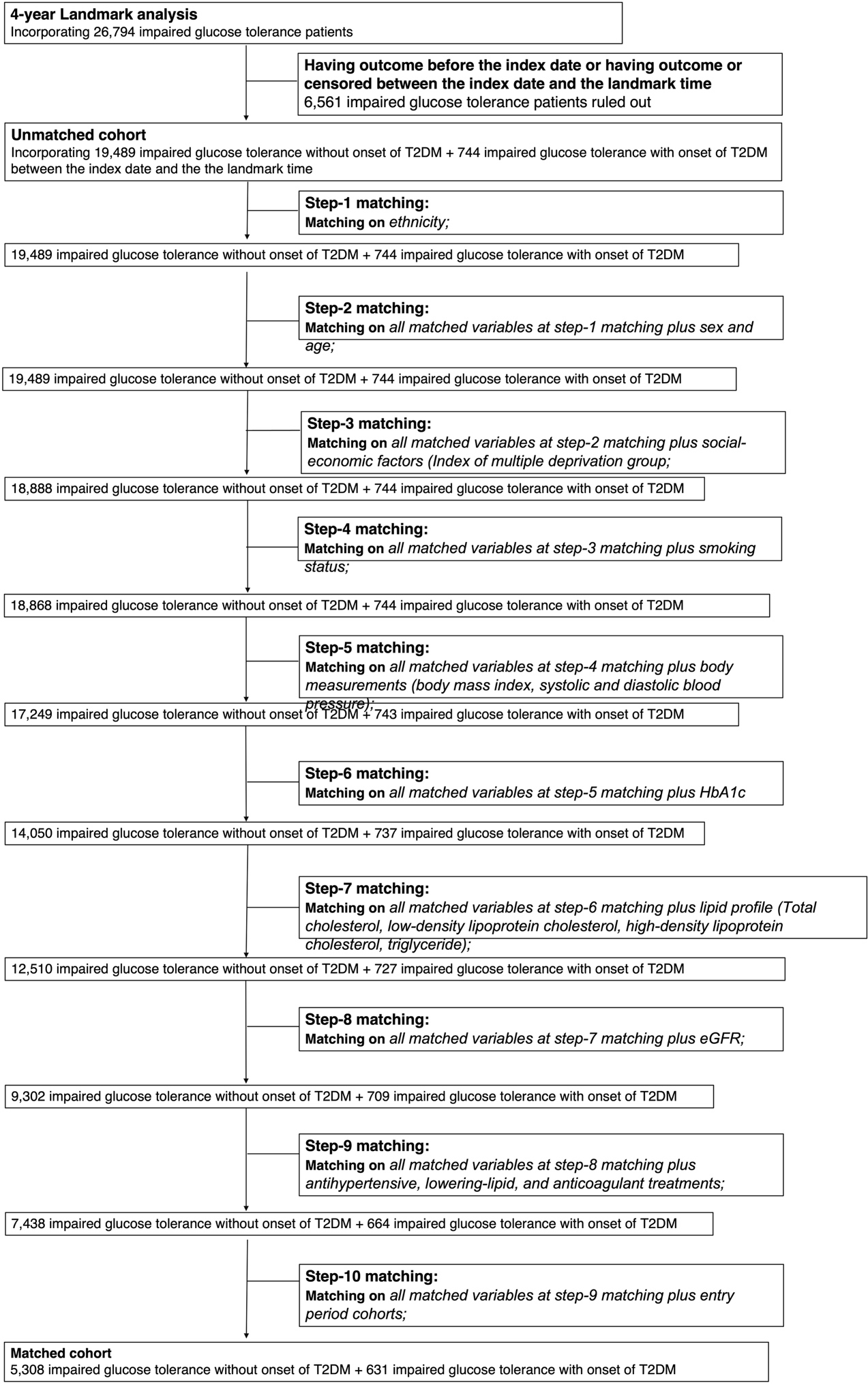


**Figure S6**. Workflow charts for matching process (5-year landmark analysis)


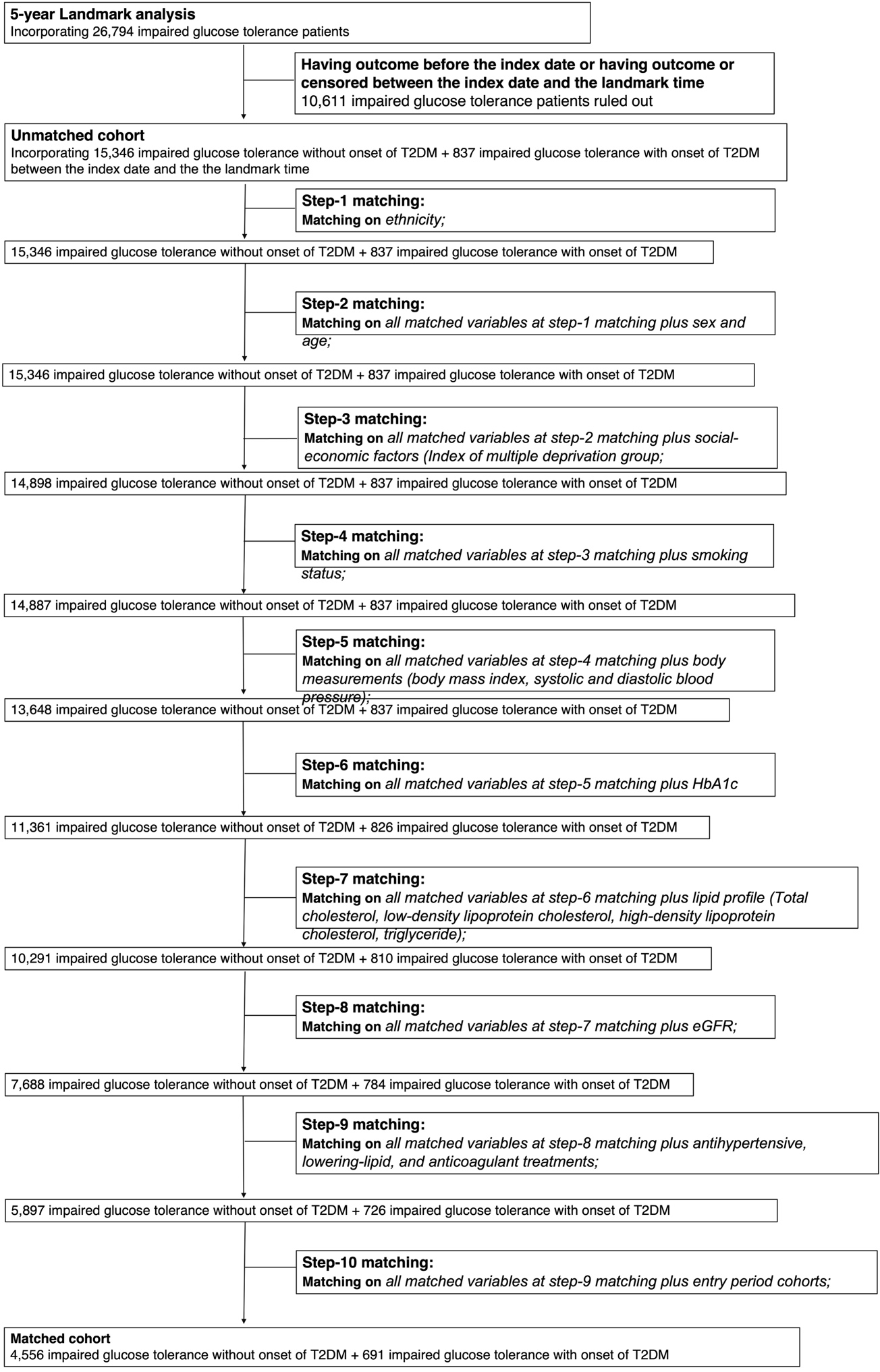


**Figure S7**. Actual Age by Temporal Median (IQR) Years at Baseline, Landmark Time points, and Follow-Up Time Points.

**
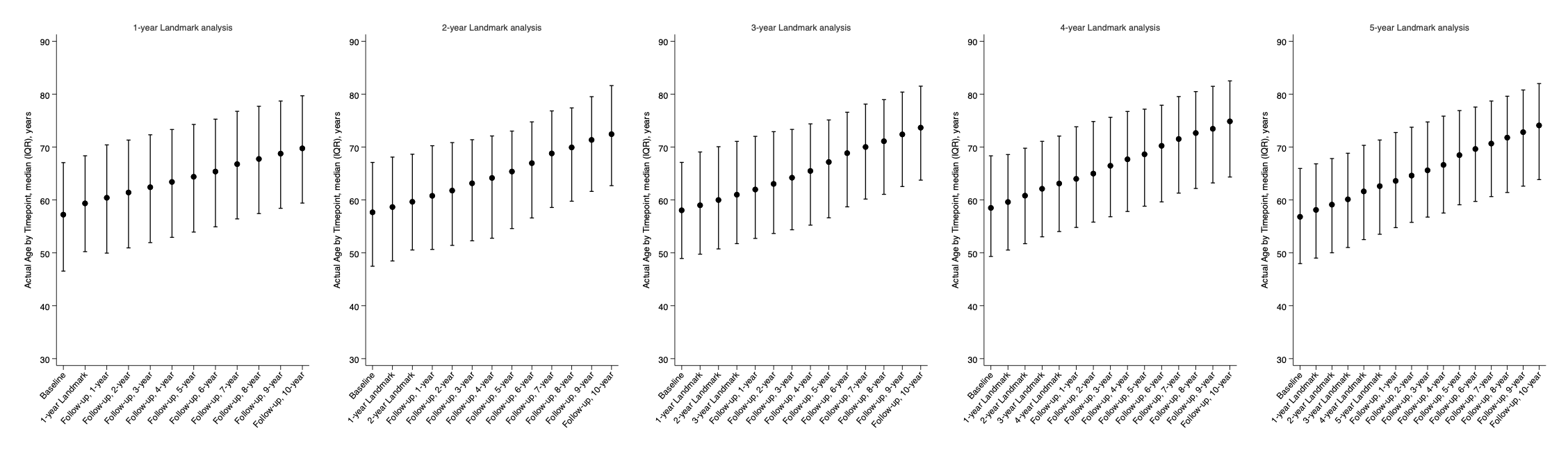
**

**Figure S8**. Adjusted incidence rate ratios for 5-year incidence rate of dementia between patients with impaired glucose tolerance with and without onset of type 2 diabetes

*Model (i) weighted for ethnicity; model (ii) weighted for all adjusted variables in model (i) plus age and sex; model (iii) weighted for all adjusted variables in model (ii) plus IMD group; model (iv) weighted for all adjusted variables in model (iii) plus smoking status; model (v) weighted for all adjusted variables in model (iv) plus body measurements (body mass index, systolic and diastolic blood pressure); model (vi) weighted for all adjusted variables in model (v) plus baseline HbA1c; model (vii) weighted for all adjusted variables in model (vi) plus baseline lipid profile (total cholesterol, low-density lipoprotein cholesterol, high-density lipoprotein cholesterol, and triglyceride); model (viii) weighted for all adjusted variables in model (vii) plus eGFR; model (ix) weighted for all adjusted variables in model (viii) plus antihypertensive, lowering lipid and anticoagulant treatment; model (x) weighted for all adjusted variables in model (ix) plus entry cohorts; The log-scale for Y-axis (incidence rate ratio) was applied.*

**Figure S9**. Adjusted incidence rate ratios for 10-year incidence rate of dementia between people with impaired glucose tolerance with and without onset of type 2 diabetes

*Model (i) weighted for ethnicity; model (ii) weighted for all adjusted variables in model (i) plus age and sex; model (iii) weighted for all adjusted variables in model (ii) plus IMD group; model (iv) weighted for all adjusted variables in model (iii) plus smoking status; model (v) weighted for all adjusted variables in model (iv) plus body measurements (body mass index, systolic and diastolic blood pressure); model (vi) weighted for all adjusted variables in model (v) plus baseline HbA1c; model (vii) weighted for all adjusted variables in model (vi) plus baseline lipid profile (total cholesterol, low-density lipoprotein cholesterol, high-density lipoprotein cholesterol, and triglyceride); model (viii) weighted for all adjusted variables in model (vii) plus eGFR; model (ix) weighted for all adjusted variables in model (viii) plus antihypertensive, lowering lipid and anticoagulant treatment; model (x) weighted for all adjusted variables in model (ix) plus entry cohorts; The log-scale for Y-axis (incidence rate ratio) was applied.*

**Figure-S10.** Overall and stratified adjusted incidence rate ratios for the 5-year and 10-year incidence rate of vascular dementia on the 5-year landmark using the final tapered matched models


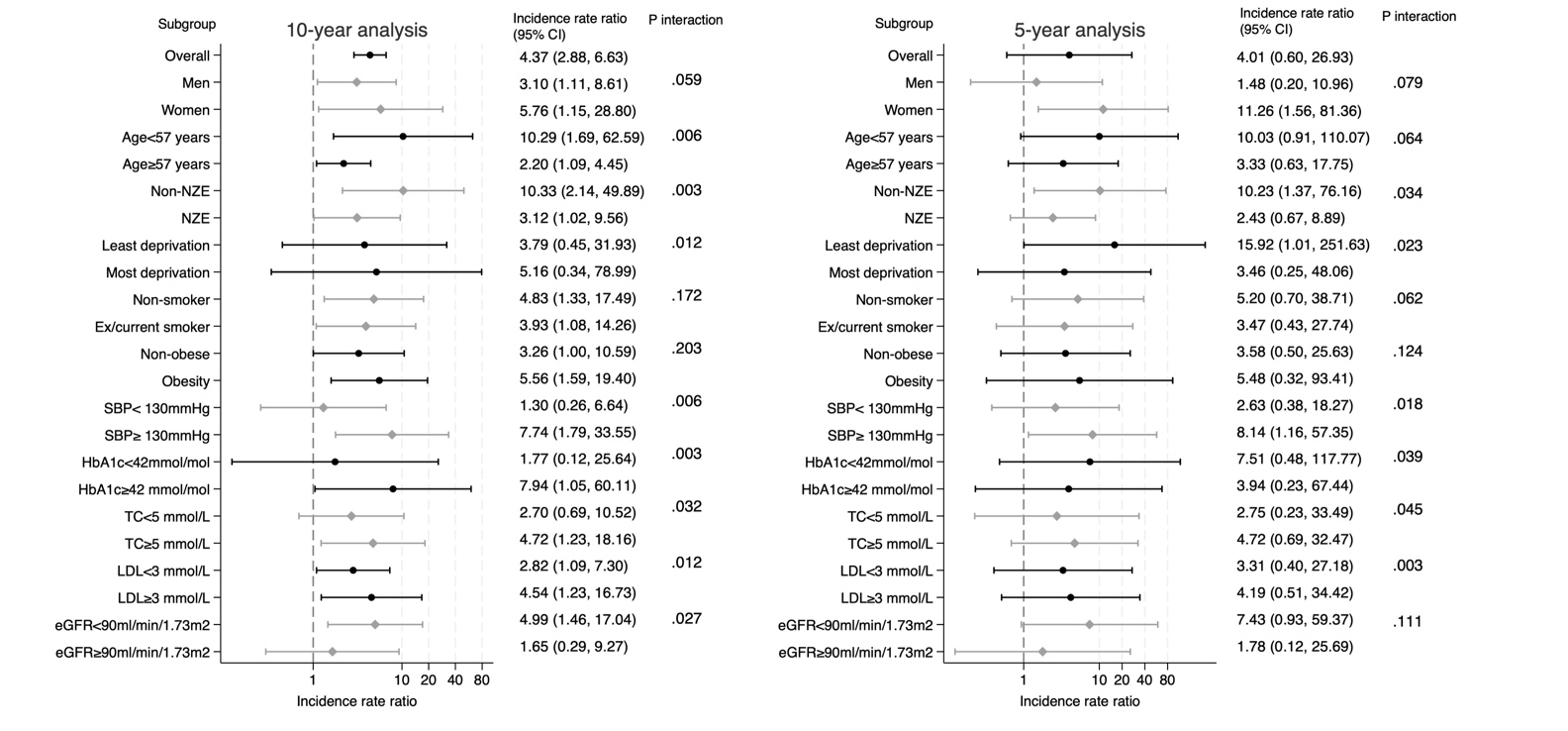

Supplement: Supplementary file 1 — Supporting Information [file ALZ-20-4423-s002.docx]
